# Adaptation of *Lactobacillus casei* Zhang to Gentamycin Involves an Alkaline Shock Protein

**DOI:** 10.3389/fmicb.2017.02316

**Published:** 2017-11-23

**Authors:** Wenyi Zhang, Huiling Guo, Chenxia Cao, Lina Li, Lai-Yu Kwok, Heping Zhang, Zhihong Sun

**Affiliations:** Key Laboratory of Dairy Biotechnology and Engineering, Ministry of Education, Key Laboratory of Dairy Products Processing, Ministry of Agriculture, Inner Mongolia Agricultural University, Hohhot, China

**Keywords:** *Lactobacillus casei* Zhang, proteomic analysis, gentamycin, alkaline shock protein, *asp*23

## Abstract

*Lactobacillus* (*L. casei*) Zhang is a koumiss-originated probiotic strain, which was used as a model in a long-term antibiotics-driven evolution experiment to reveal bacterial evolutionary dynamics; and we isolated gentamycin-resistant *L. casei* Zhang descendents. To decipher the gentamycin resistance mechanism, here we cultivated the parental *L. casei* Zhang and its descendent cells in an antibiotics-containing environment to compare their global protein expression profiles using the iTRAQ-based proteomic approach. A total of 72 proteins were significantly up-regulated (>2.0-fold, *P* < 0.05), whilst 32 proteins were significantly down-regulated <−2.0-fold, *P* < 0.05) in the descendent line. The gentamycin-resistant descendent line showed elevated expression in some carbohydrates, amino acids, and purine metabolic pathways. Several stress-related proteins were also differentially expressed. Among them, one alkaline shock protein, *asp*23, was up-regulated most in the gentamycin-resistant strain (21.9-fold increase compared with the parental strain). The *asp*23 gene disruption mutant was significantly more sensitive to gentamycin compared with the wild type, suggesting an important role of this gene in developing the gentamycin-resistant phenotype in *L. casei*. Our report has described the adaptation of a probiotic strain that has acquired antibiotics resistance through long-term antibiotics exposure at the proteome level, and we revealed a novel mechanism of gentamycin resistance.

## Introduction

*Lactobacillus* (*L. casei*) is the dominant species within the genus *Lactobacillus* found in koumiss, a naturally fermented dairy product (Wu et al., 2009a). Besides, it has been detected in corn silage, wine, pickle, human gastrointestinal tracts, blood, yogurt, and cheese (Cai et al., 2007; Bao et al., 2016). The wide ecological distribution of *L. casei* reflects its flexibility in metabolizing carbohydrates (Zhang et al., 2010), and thus it is commonly used in food industry. The species *Lactobacillus casei* has been used both as starter cultures and as food additives for improving food texture properties (Dantas et al., 2016). Some strains are considered as probiotics bacteria due to their beneficial effects, such as antibacterial, antioxidative, and immunomodulatory properties (Ya et al., 2008; Zhang et al., 2014b; Wang et al., 2016). Meanwhile, the increasing availability of the whole genome sequences of representative *L. casei* strains and genetic tools for creating recombinant *Lactobacillus* has largely facilitated genetic and functional studies, leading to remarkable progress in our understanding of the cell biology of these bacteria (Xu and Kong, 2013; Lu et al., 2016).

Antibiotic resistance of bacteria is an increasingly serious public health threat (Normark and Normark, 2002). This is especially critical for pathogenic bacteria that can rapidly become antibiotic-resistant in response to clinical application of antibiotics (Arias and Murray, 2015). It is unlikely that the probiotics used in food industry face a similar situation due to low exposure to antibiotics within the food matrix. Moreover, strict regulations must be followed by the food industry to avoid unnecessary use of antibiotics (Lara et al., 2006). The dairy industry even routinely monitors raw milk against antibiotics contamination because such contaminants would suppress or even kill lactic acid bacteria (LAB) and subsequently affect milk fermentation. The addition of bacteriocins or bacteriocin-producing bacteria has been explored as a method of food preservation, but it is not a wide-spread practice (Galvez et al., 2007). However, owing to the imminent global health concern of bacterial antibiotic resistance, the use of probiotics has been proposed as a valuable adjunct or even alternative to antibiotic therapy in clinical practice due to both their health-promoting properties and intrinsic bacteriocidal effects (Boyanova and Mitov, 2012; Reid, 2017). Several clinical trials supported the use of probiotics in the management of acute gastroenteritis and antibiotic-associated diarrhea (Katz, 2010). In these situations, probiotics may be directly exposed to antibiotics, sometimes even at a high concentration. Thus, it would be crucial to characterize the adaptation of probiotics to antibiotics stress.

*Lactobacillus casei* Zhang is a probiotic strain isolated from koumiss (Zhang et al., 2014a, 2015). We previously performed a long-term evolution experiment using *L. casei* Zhang as a model to reveal bacterial evolutionary dynamics under antibiotic stress; and we generated gentamycin-resistant *L. casei* Zhang descendents (Wang et al., 2017). During the experiment, the bacteria developed resistance to gentamycin gradually, and the accumulation of genome point mutation stopped shortly after the descendent bacteria reached the maximum bacterial fitness. To decipher the mechanism of the resistance phenotype, here we compared the global protein expression profiles between the parental *L. casei* Zhang and its descendent cells grown under antibiotic selection force using the iTRAQ-based proteomic approach. Furthermore, we validated the expression of one selected differentially expressed protein using parallel reaction monitoring (PRM), followed by disrupting the corresponding gene to verify its function in the development of gentamycin-resistant phenotype of *L. casei* Zhang.

## Materials and methods

### Bacterial isolates and culture conditions

*Lactobacillus casei* Zhang-G-1200 was isolated from a long-term laboratory-based evolution experiment performed in our laboratory (Wang et al., 2017). The strain Zhang-G-1200 exhibited a higher resistance to gentamycin compared with the parental cells (minimum inhibitory concentration, MIC, of 32 μg/mL for Zhang-G-1200 vs. 2 μg/mL for the parental strain). The acquisition of resistance was a result of multigenerational and stepwise increase during the prolonged cultivation under gentamycin stress. Growth curves of the 2 strains were constructed based on optical density (OD) measurement every 2 h along 30-h fermentation in LSM supplemented with 1 μg/mL gentamycin. Meanwhile, changes in pH and viable counts were determined. All analyses were performed in triplicate.

The bacterial strain, *Escherichia* (*E. coli*) DH5α, was used as a host for standard cloning procedures. It was propagated aerobically in Luria Bertani broth at 37°C. Chloramphenicol (10 μg/ml for both *E. coli* and *L. casei* Zhang-G-1200) and erythromycin (250 and 5 μg/ml for *E. coli* and *L. casei* Zhang-G-1200, respectively) were used for selecting genetically modified bacterial clones.

### Sample preparation, protein digestion and iTRAQ labeling

Both the parental and *L. casei* Zhang-G-1200 cells were collected after 24 h of cultivation in LSM supplemented with 1 μg/mL gentamycin. Four biological replicates were prepared for each of the *L. casei* Zhang and the Zhang-G-1200 strains. Cells were harvested by centrifugation, followed by washing with phosphate buffered saline for 4 times. One milliliter of lysis buffer was added to each sample, followed by sonication on ice and centrifugation at 13,000 rpm for 10 min at 4°C. The protein concentration of sample supernatants was determined by using the bicinchoninic acid protein assay.

Hundred microgram protein was transferred to a new tube and adjusted to a protein concentration of 1 μg/μL with 100 mM triethylammonium bicarbonate (TEAB). Five microliters of 200 mM DTT were added and incubated at 55°C for 1 h, then 5 μL of 375 mM iodoacetamide was added to the sample and incubated for 30 min at room temperature in the dark. For each sample, proteins were precipitated with ice-cold acetone before re-dissolving in 20 μL TEAB. Proteins were then tryptically digested with sequencing grade modified trypsin (Promega, Madison, WI), and the resultant peptide mixture was labeled using reagents from the iTRAQ reagents kit.

### High pH reverse phase separation by UPLC

The peptide mixture was redissolved in buffer A (buffer A: 10 mM ammonium formate in water, pH 10, adjusted with ammonium hydroxide) and then fractionated by high pH separation using an Aquity UPLC system (Waters Corporation, Milford, MA) connected to a reverse phase column (BEH C18 column, 2.1 × 150 mm, 1.7 μm, 300 Å, Waters Corporation, Milford, MA). High pH separation was done using a linear gradient, starting from 0% B to 45% B in 35 min (B: 10 mM ammonium formate in 90% acetonitrile, pH 10.0, adjusted with ammonium hydroxide). The column flow rate and temperature were maintained at 250 μL/min and 45°C, respectively. Sixteen fractions were collected, and each fraction was dried in a vacuum concentrator prior to the next step.

### Low pH nano-HPLC-MS/MS analysis

The dried fractions were re-suspended in a solution made of solvent C and D (C: water with 0.1% formic acid; D: acetonitrile with 0.1% formic acid), separated by nano LC, and analyzed by on-line electrospray tandem mass spectrometry. The experiments were performed on a Nano Aquity UPLC system (Waters Corporation, Milford, MA) connected to a quadrupole-Orbitrap mass spectrometer (Q-Exactive) (Thermo Fisher Scientific, Bremen, Germany) equipped with an online nano-electrospray ion source. Eight microliters of peptide sample were loaded onto the trap column (Thermo Scientific Acclaim PepMap C18, 100 μm × 2 cm), with a flow of 10 μl/min for 3 min, and subsequently separated on an analytical column (Acclaim PepMap C18, 75 μm × 25 cm) with a linear gradient, from 5% D to 30% D in 95 min. The column was re-equilibrated to the initial conditions for 15 min. The column flow rate and temperature were maintained at 300 nL/min and 45°C, respectively. An electrospray voltage of 2.0 kV was used against the inlet of the mass spectrometer.

The Q Exactive Hybrid Quadrupole-Orbitrap Mass Spectrometer was operated in the data-dependent mode to switch automatically between MS and MS/MS acquisition. Survey full-scan MS spectra (m/z 350–1,600) were acquired with a mass resolution of 70 K, followed by 15 sequential high-energy-collisional-dissociation (HCD) MS/MS scans with a resolution of 17.5 K. In all cases, one micro-scan was recorded using dynamic exclusion of 30 s, with an MS/MS fixed first mass of 100.

### Database searching and data analysis

Tandem mass spectra were extracted by the Proteome Discoverer software (Thermo Fisher Scientific, version 1.4.0.288). The mass profiles generated from all samples were searched with Mascot (Matrix Science, London, UK; version 2.3) against the NCBI database (Taxonomy: *L. casei* Zhang). We used the percolator algorithm <1% to control peptide level false discovery rates. Only unique peptides were used for protein quantification and the normalization on protein median was applied to correct any experimental bias. The minimum number of proteins that must be observed was set to 1,000. Students'*t*-tests were performed with the software package R, with *p* < 0.05 considered statistically significant. A 2.0-fold change was used as the threshold for selection of differentially regulated proteins. All regulated proteins were distributed over clusters of orthologous genes (COGs) and searched against the Kyoto Encyclopedia of Genes and Genomes database.

### Validation of expression level of an alkaline shock protein by PRM

Based on the results of proteomic analysis, 1 protein (the alkaline shock protein, coded by the gene LCAZH_0227) was selected for PRM analysis. This protein was most up-regulated among all differentially expressed proteins.

Experiments were performed on a Q Exactive mass spectrometer coupled with Easy-nLC1200. Peptide mixtures were separated by C18-reversed phase chromatography on an Easy column (75 μm × 25 cm), and the analytical separation was run for 90 min using a linear gradient of ACN/FA 2%/0.1% (Solvent A) and ACN/FA 80%/0.1% (Solvent B) at a flow rate of 300 nL/min. The gradient programme was run as follows: 5% B at 1 min, ramping to 23% B at 41 min, 29% B at 51 min, rapid ramping to 100% B over 8 min and holding 100% B for 6 min before returning to initial condition of 5% B. The column was re-equilibrated to 5% B for 30 min after each run. All samples were analyzed using a multiplexed PRM method based on a scheduled inclusion list containing the target precursor ions representing the standard peptides. The full scan event was collected at m/z 300–1300, an Orbitrap resolution of 70,000, the automatic gain control target at 3e6, and the maximum fill time at 20 ms. Every full scan was followed by 10 PRM scans at a resolution of 17,500 with an isolation window: 2.0 m/z, an AGC value of 5e5, the maximum fill time of 100 ms, and a normalized collision energy of 28 in a higher-energy c-trap dissociation (HCD) cell.

PRM data analysis and data integration were performed with the Skyline Software. Routine assessment of instrument and chromatographic performance was done with a quality control (QC) standard consisting of all synthetic peptides, which was prepared at a concentration of 20 fmol/μl in 0.1% formic acid. Every sample was injected for three times, the peak areas of the target peptides were extracted using Skyline, peak peaking was manually checked and corrected in accordance to the retention time, transitions, mass accuracy, and MS/MS spectra. At least 3 transitions for each peptide were extracted from the PRM data. Peptides were quantified by summing the peak areas under curve (AUC) of each transition. Peptide abundance was normalized based on the total ion current (TIC) extracted from the full scan acquisition for each run. Proteins were quantified by summing the abundances of the selected peptides, and the accurate protein quantities had to match those of the synthetic standard peptides.

### Construction and analysis of an alkaline shock protein gene disruption mutant

The gene LCAZH_0227 (*asp*23) was selected for target disruption. Plasmids and primers used for gene disruption are listed in Table 1. The mutant line was constructed by using a cre-lox-based system originally developed by Lambert et al. (2007). Briefly, the upstream (amplified with the primers 0227upF and 0227upR,) and downstream (amplified with 0227downF and 0227downR) fragments of the LCAZH_0227 gene were PCR amplified from the genomic DNA of *L. casei* Zhang-G-1200. The fragments were cloned between the *Sal*I-HF or *Pme*I and *Ecol* 53KI or *Bgl*II restriction sites of the suicide vector pNZ5319 to form the recombinant mutagenesis vector, pNZ5319-0227 Up-Down. To inactivate the LCAZH_0227 gene, pNZ5319-0227 Up-Down was introduced into *L. casei* Zhang-G-1200 by electroporation. Chloramphenicol-resistant transformants were selected and replica plated to check for an erythromycin-sensitive phenotype. Candidate double-crossover mutant clones were identified by PCR, and correct integration of the lox66-P32-cat-lox71 cassette into the genome was further verified by PCR using the primers 0227upF or catR and catF or 0227downR. To excise the P32-cat selectable marker cassette, the cre expression plasmid pMSPcre was transformed into the 0227::lox66-P32-cat-lox71 gene replacement mutant. The Cre-mediated recombination and correct excision of the P32-cat cassette were checked by PCR using primers spanning the recombination loci (0227upF and 0227downR). The pMSPcre vector was cured from *L. casei* Zhang-G-1200 Δ0227 colonies by growth without erythromycin selection pressure. Additionally, PCR products were confirmed by sequencing when necessary.

**Table 1:** Strains, primers, and plasmids used for constructing the gene disruption mutant.

**Strains, plasmids, and primers**	**Description or primer sequence^a^**	**Reference or source**
**STRAINS**		
*E. coli* DH5α	Cloning host	This study
*L. casei* Zhang	Isolated from home-made koumiss in Inner Mongolia, China	Wu et al., 2009a
*L. casei* Zhang-G-1200	*L. casei* Zhang propagated in LSM broth containing gentamycin 1μg/mLfor 6 months	Wang et al., 2017
*L. casei* Zhang-G-1200-0227::*lox66*-P32-*cat-lox71*	Derivative of *L. casei* Zhang-G-1200 containing a *lox66*-P32-*cat-lox71* replacement of LCAZH_0227	This study
*L. case*i Zhang-G-1200-Δ0227	Derivative of *L. casei* Zhang-G-1200-0227::*lox66*-P32-*cat-lox71* containing a *lox72* replacement of LCAZH_0227	This study
**PLASMIDS**		
pNZ5319	Cm^r^Em^r^; containing *lox66*-P32-*cat-lox71* cassette for multiple gene replacement in Gram-positive bacteria	Lambert et al., 2007
pNZ5319-0227Up-Down	Cm^r^Em^r^; pNZ5319 derivative containing homologous regions upstream and downstream of LCAZH_0227	This study
pMSPcre	Em^r^; expression of *cre*	Unpublished
**PRIMERS**		
0227upF	5′-ACGCGTCGACGTCGCCTGTCTCGGTATTCCTGTG-3′	This study
0227upR	5′-AGCTTTGTTTAAACGGCGCGCCGGTATTGATGCCAGCGTTT-3′	This study
0227downF	5′-GGGTTTGAGCTCTTGCCAGTTCAGTCGTT-3′	This study
0227downR	5′-GAAGATCTTCTCATTTGCCTCCCTTAT-3′	This study
85	5′-GTTTTTTTCTAGTCCAAGCTCACA-3′	Lambert et al., 2007
87	5′-GCCGACTGTACTTTCGGATCCT-3′	Lambert et al., 2007
CatF	5′-TCAAATACAGCTTTTAGAACTGG-3′	Lambert et al., 2007
CatR	5′-ACCATCAAAAATTGTATAAAGTGGC-3′	Lambert et al., 2007
EryintF	5′-CGATACCGTTTACGAAATTGG-3′	Lambert et al., 2007
EryintR	5′-CTTGCTCATAAGTAACGGTAC-3′	Lambert et al., 2007

a *The restriction sites in the primer sequences are underlined*.

The growth performance and gentamycin-resistant phenotype of the mutant strain was evaluated by viable counts, OD measurements, and MIC of gentamycin (Guo et al., 2017). Phenotypic differences between the wild-type and mutant strains were determined statistically by Student's *t*-test.

## Results

### Growth performance of the parental and *L. casei* Zhang-G-1200 strains

**Figure 1:**
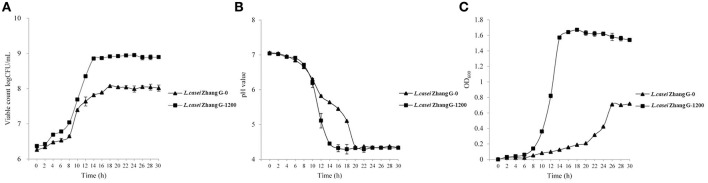
Growth curves of the parental and *L. casei* Zhang-G-1200 strains based on viable counts **(A)**, pH values **(B)** and OD values **(C)** in gentamycin-containing LSM.

The growth of the two bacterial lines in the presence of gentamycin was monitored. Growth curves were plotted based on viable counts, pH values, and OD values. As shown in Figures 1A–C, the growth performance of *L. casei* Zhang-G-1200 was completely different from that of the parental line. The viable counts of *L. casei* Zhang-G-1200 increased more rapidly than that of the parental line, reaching a maximum cell density of 9.05 × 10^7^ cfu/mL after 20 h (Figure 1A). Meanwhile, the pH of the LSM medium inoculated with *L. casei* Zhang-G-1200 dropped much faster than that of the parental line, indicating a higher fermentation rate (Figure 1B).

### Up-regulated *L. casei* Zhang-G-1200 proteins grown in gentamycin-containing medium

**Figure 2:**
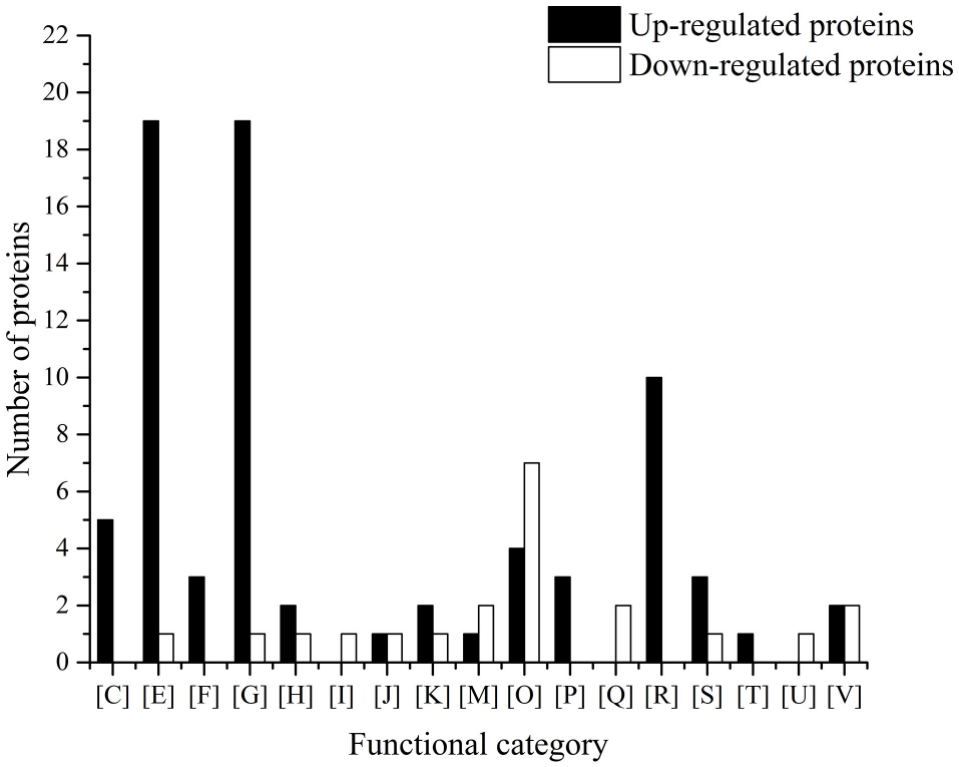
Proteomic profiles of differentially expressed proteins of the parental *L. casei* Zhang G-0 and gentamycin-resistant *L. casei* Zhang-G-1200 strains. Clusters of orthologous groups (COG) functional categories: [C], Energy production and conversion; [E], Amino acid transport and metabolism; [F] Nucleotide transport and metabolism; [G], Carbohydrate transport and metabolism; [H], Coenzyme transport and metabolism; [I], Lipid transport and metabolism; [J], Translation, ribosomal structure and biogenesis; [K], Transcription; [L], Replication, recombination and repair; [M], Cell wall/membrane/envelope biogenesis; [O], Posttranslational modification, protein turnover, chaperones; [P], Inorganic ion transport and metabolism; [Q], Secondary metabolites biosynthesis, transport and catabolism; [R], General function prediction only; [S], Function unknown; [T], Signal transduction mechanisms; [V], Defense mechanisms.

A total of 72 proteins were significantly up-regulated (>2.0-folds, *P* < 0.05) in *L. casei* Zhang-G-1200 compared with the parental strain (Table 2). Most of these proteins could be assigned to one of the functional COGs categories (Figure 2), with 26.3% of these proteins involved in carbohydrate transport and metabolism [G] and another 25.3% of them involved in amino acid transport and metabolism [E].

**Table 2:** Up-regulated proteins of *L. casei* Zhang-G-1200 compared with its original strain in the presence of the gentamycin.

**Locus**	**Function**	**Fold change**
**CARBOHYDRATE TRANSPORT AND METABOLISM**		
LCAZH_0394	Hypothetical protein	2.07
LCAZH_0395	Mannose-6-phosphate isomerase	3.06
LCAZH_0403	PTS system mannose/fructose/N-acetylgalactosamine-specific transporter subunit IIB	2.23
LCAZH_0503	Sugar phosphate isomerase/epimerase	2.19
LCAZH_0604	PTS system galactitol-specific transporter subunit IIB	2.1
LCAZH_0605	PTS system galacitol transporter subunit EIIC	2.22
LCAZH_1336	Tagatose-6-phosphate kinase	2.41
LCAZH_1768	Beta-glucosidase/6-phospho-beta-glucosidase/beta-galactosidase	2.13
LCAZH_1771	PTS system cellobiose-specific transporter subunit IIA	2.2
LCAZH_1772	PTS system cellobiose-specific transporter subunit IIB	2.61
LCAZH_2151	beta-glucosidase/6-phospho-beta-glucosidase/beta-galactosidase	2.14
LCAZH_2624	PTS system fructose-specific transporter subunit IIB	2.36
LCAZH_2633	PTS system galactitol transporter subunit IIB	2.97
LCAZH_2645	Hypothetical protein	2.53
LCAZH_2648	PTS system galacitol transporter subunit EIIB	5.79
LCAZH_2649	PTS system galacitol transporter subunit EIIA	3.96
LCAZH_2653	Trehalose-6-phosphate hydrolase	4.66
LCAZH_2725	Transaldolase	4.56
LCAZH_2895	PTS system mannitol-specific transporter subunit IIBC	3.96
**AMINO ACID TRANSPORT AND METABOLISM**		
LCAZH_0084	Tryptophan synthase subunit alpha	2.25
LCAZH_0107	Tetrahydrodipicolinate N-succinyltransferase	2.25
LCAZH_0201	Oligopeptide ABC transporter periplasmic protein	2.04
LCAZH_0418	Amino acid ABC transporter ATP-binding protein	2.14
LCAZH_0500	Amino acid transporter	2.59
LCAZH_0506	Shikimate 5-dehydrogenase	2.08
LCAZH_1596	Oligopeptide ABC transporter periplasmic protein	2.47
LCAZH_1682	Lactoylglutathione lyase-like lyase	2.22
LCAZH_1886	Oligopeptide ABC transporter periplasmic protein	2.92
LCAZH_1980	Branched-chain amino acid aminotransferase/4-amino-4-deoxychorismate lyase	2.04
LCAZH_2023	Dipeptide/oligopeptide/nickel ABC transporter ATPase	2.21
LCAZH_2024	Dipeptide/oligopeptide/nickel ABC transporter permease	2.45
LCAZH_2025	Dipeptide/oligopeptide/nickel ABC transporter permease	3.27
LCAZH_2026	Oligopeptide ABC transporter periplasmic protein	2.68
LCAZH_2111	Homoserine dehydrogenase	2.36
LCAZH_2302	Aminopeptidase	6.77
LCAZH_2518	NADPH-dependent glutamate synthase subunit beta-like oxidoreductase	3.52
LCAZH_2519	Glutamate synthase domain 3	2.54
LCAZH_2851	Polar amino acid ABC transporter ATPase	2.23
**ENERGY PRODUCTION AND CONVERSION**		
LCAZH_0188	Acetate kinase	2.17
LCAZH_1301	Acetoin/pyruvate dehydrogenase complex, E2 component, dihydrolipoamide succinyltransferase	2.05
LCAZH_1302	Acetoin/pyruvate dehydrogenase complex, E3 component, dihydrolipoamide dehydrogenase	2.13
LCAZH_1396	Pyruvate-formate lyase	3.75
LCAZH_2375	Fumarase	2.1
**NUCLEOTIDE TRANSPORT AND METABOLISM**		
LCAZH_1739	Folate-dependent phosphoribosylglycinamide formyltransferase PurN	2.5
LCAZH_1740	Phosphoribosylaminoimidazole (AIR) synthetase	2.23
LCAZH_1743	Phosphoribosylformylglycinamidine (FGAM) synthase, glutamine amidotransferase domain	2.21
**CELL WALL/MEMBRANE/ENVELOPE BIOGENESIS**		
LCAZH_0447	Conjugated bile salt hydrolase-like amidase	5.04
**TRANSCRIPTION**		
LCAZH_1410	GNAT family acetyltransferase	2.42
LCAZH_2210	Transcriptional regulator	3.18
**COENZYME TRANSPORT AND METABOLISM**		
LCAZH_1463	Lipoate-protein ligase A	2.05
**TRANSLATION, RIBOSOMAL STRUCTURE AND BIOGENESIS**		
LCAZH_1880	Acetyltransferase	3.78
**POSTTRANSLATIONAL MODIFICATION, PROTEIN TURNOVER, CHAPERONES**		
LCAZH_0279	ADP-ribosylglycohydrolase	2.51
LCAZH_1344	Chaperone ClpB	3.68
LCAZH_1380	Peptide methionine sulfoxide reductase	2.23
LCAZH_1398	Pyruvate-formate lyase-activating enzyme	2.88
**DEFENSE MECHANISMS**		
LCAZH_1217	Multidrug ABC transporter ATPase	6.01
**GENERAL FUNCTION PREDICTION ONLY**		
LCAZH_0294	Alpha/beta hydrolase	2.64
LCAZH_0305	NAD(FAD)-dependent dehydrogenase	2.14
LCAZH_0638	ABC transporter periplasmic protein	2.06
LCAZH_0641	ABC transporter permease	2.3
LCAZH_0642	ABC transporter ATPase	2.15
LCAZH_1865	Dinucleotide-binding enzyme	2.05
LCAZH_2372	Oxidoreductase	2.53
LCAZH_2373	Short-chain alcohol dehydrogenase	3.71
**FUNCTION UNKNOWN**		
LCAZH_0227	Alkaline shock protein	21.93
LCAZH_2030	Hypothetical protein	3.57
LCAZH_2301	Putative integral membrane protein	3.32
LCAZH_2056	Hypothetical protein	2.3
LCAZH_2222	Hypothetical protein	3.29
LCAZH_1464	Hypothetical protein	3.26
LCAZH_1898	Hypothetical protein	2.42
LCAZH_0186	Hypothetical protein	2.34

Over half (6 out of 10) of the up-regulated proteins were associated with the COGs category of carbohydrate transport and metabolism [G], which were the phosphotransferase system (PTS)-related components located within 3 different operons. According to the Transporter Classification Database (TCDB), 4 of these differentially regulated proteins belonged to the PTS Galactitol family, including the galacitol transporter subunits EIIA (LCAZH_2649), EIIB (LCAZH_2648), IIB (LCAZH_0604), and EIIC (LCAZH_0605), while the other 2 belonged to the PTS Lactose-N, N'-Diacetylchitobiose-β-glucoside family, namely the cellobiose-specific transporter subunits IIA (LCAZH_1771) and IIB (LCAZH_1772).

Another group of significantly up-regulated proteins was responsible for the uptake of oligopeptides (LCAZH_2023-LCAZH_2026). This genomic cluster coded for the ATPase and 2 permease components of a dipeptide/oligopeptide/nickel ABC transporter, as well as an oligopeptide ABC transporter periplasmic protein. Similar to the Opp system identified in other LAB genomes (Chen et al., 2015), another gene that coded for the ATP-binding subunit of the oligopeptide ABC transporter was found immediately downstream of the oligopeptide ABC transporter periplasmic protein of the genome of *L. casei* Zhang (Zhang et al., 2010). Besides, several other oligopeptide (LCAZH_0201, LCAZH_1596, LCAZH_1886, and LCAZH_2851) and amino acid (LCAZH_0500 and LCAZH_0418) transporter components, as well as subunits for *de novo* syntheses of tryptophan (LCAZH_0084) and glutamate (LCAZH_2518 and LCAZH_2519), were identified.

Other up-regulated proteins included those relating to purine biosynthesis (LCAZH_1739, LCAZH_1740, and LCAZH_1743) and stress response (LCAZH_1344 and LCAZH_0227). Notably, the alkaline shock protein (LCAZH_0227) was the most significantly up-regulated protein with a 21.9-fold increase in its expression compared with the parental line, suggesting its important role in gentamycin adaptation.

### Down-regulated *L. casei* Zhang-G-1200 proteins grown in gentamycin-containing medium

A total of 36 proteins were significantly down-regulated (<−2.0-folds, *P* < 0.05) in *L. casei* Zhang-G-1200 compared with the parental strain (Table 3). According to the COGs functional classification, 33.3% of these proteins were involved in posttranslational modification, protein turnover, chaperones metabolism [G] (Figure 2). Indeed, most of them were stress-responsive proteins, including the protease subunit of ATP-dependent *Clp* protease (LCAZH_0907), the molecular chaperones *Grp*E, *Gro*EL, and *HSP*20-2 (LCAZH_1553, LCAZH_2207, and LCAZH_2811), the co-chaperonin *Gro*ES (LCAZH_2208), and the *Clp* protease/*Dna*K/*Dna*J chaperone ATP-binding subunit (LCAZH_1753), while the others were mainly hypothetical proteins of unknown functions.

**Table 3:** Down-regulated proteins of *L. casei* Zhang-G-1200 compared with its original strain in the presence of the gentamycin.

**Locus**	**Function**	**Fold change**
**CARBOHYDRATE TRANSPORT AND METABOLISM**		
LCAZH_2698	Fructose/tagatose bisphosphate aldolase	−3.3
**AMINO ACID TRANSPORT AND METABOLISM**		
LCAZH_1424	Histidinol-phosphate/aromatic aminotransferase and cobyric acid decarboxylase	−2.51
**CELL WALL/MEMBRANE/ENVELOPE BIOGENESIS**		
LCAZH_0738	D-alanyl transfer protein	−2.1
LCAZH_2870	Glycosyltransferase	−2.22
**TRANSCRIPTION**		
LCAZH_1554	Transcriptional regulator	−2.06
**POSTTRANSLATIONAL MODIFICATION, PROTEIN TURNOVER, CHAPERONES**		
LCAZH_0497	Membrane associated subtilisin-like serine protease	−2.77
LCAZH_0907	Protease subunit of ATP-dependent Clp protease	−2.04
LCAZH_1553	Molecular chaperone GrpE	−2.09
LCAZH_1753	Clp protease/DnaK/DnaJ chaperone ATP-binding subunit	−3.28
LCAZH_2207	Molecular chaperone GroEL	−2.14
LCAZH_2208	Co-chaperonin GroES (HSP10)	−2.77
LCAZH_2811	Molecular chaperone	−5.1
**COENZYME TRANSPORT AND METABOLISM**		
LCAZH_0348	Thiamine monophosphate synthase	−2.21
**LIPID TRANSPORT AND METABOLISM**		
LCAZH_0739	D-alanyl carrier protein	−3.1
**REPLICATION**		
LCAZH_0852	50S ribosomal protein L14	−2.21
**SECONDARY METABOLITES BIOSYNTHESIS, TRANSPORT AND CATABOLISM**		
LCAZH_2835	Amidase	−2.23
**DEFENSE MECHANISMS**		
LCAZH_1927	Antimicrobial peptide ABC transporter permease	−2.34
LCAZH_1928	Antimicrobial peptide ABC transporter ATPase	−2.76
LCAZH_1179	XRE family transcriptional regulator	−4.68
**FUNCTION UNKNOWN**		
LCAZH_1052	Hypothetical protein	−2.04
LCAZH_1126	Hypothetical protein	−2.01
LCAZH_1754	Hypothetical protein	−2.03
LCAZH_0994	Hypothetical protein	−2.06
LCAZH_2341	Prebacteriocin	−2.07
LCAZH_0543	Hypothetical protein	−2.1
LCAZH_0824	Hypothetical protein	−2.22
LCAZH_1530	Hypothetical protein	−2.23
LCAZH_2528	Hypothetical protein	−2.23
LCAZH_1498	Hypothetical protein	−2.24
LCAZH_2472	Hypothetical protein	−2.6
LCAZH_1584	Hypothetical protein	−2.85
LCAZH_0616	Hypothetical protein	−2.97
LCAZH_0580	Hypothetical protein	−3.06
LCAZH_0521	Hypothetical protein	−3.97
LCAZH_2689	Hypothetical protein	−4.46
LCAZH_0113	Hypothetical protein	−4.71

### Validation of expression level of an alkaline shock protein by PRM

The expression of the alkaline shock protein (LCAZH_0227) was confirmed by PRM analysis. Fortunately, 1 unique peptide (FDDAVIAK) corresponding to this protein was found, which enabled the downstream quantification. As revealed by the analysis, the quantity of this alkaline shock protein was significantly higher in *L. casei* Zhang-G-1200 than the parental cells (2.938 ± 0.144 fmol/μg vs. 0.789 ± 0.057 fmol/μg). This result was consistent with the findings of the proteomic analysis.

### Partial reversion of gentamycin-resistant phenotype of *asp23* disruption mutant

**Figure 3:**
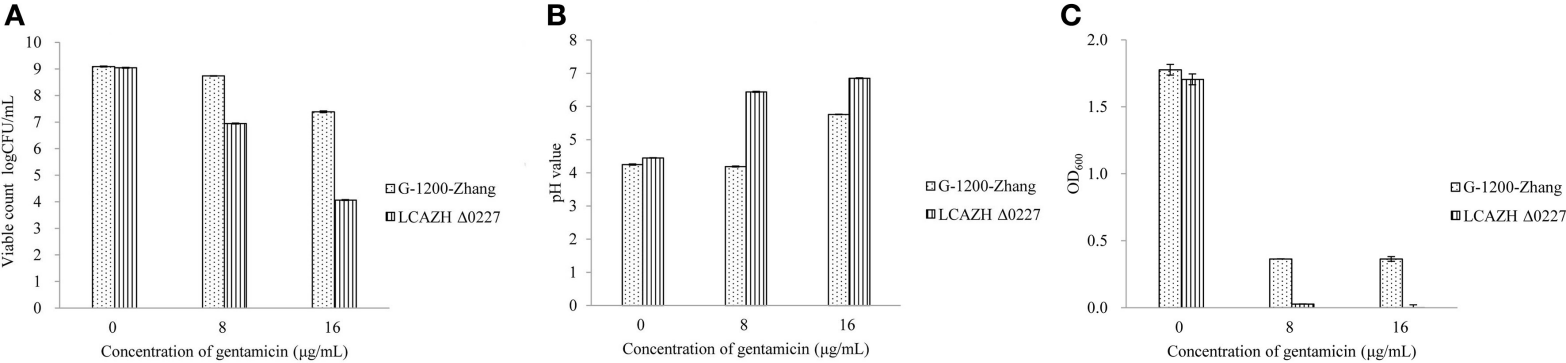
The viable counts **(A)**, pH values **(B)** and OD values **(C)** of the wild-type and mutant strains at the time of observing the minimum inhibitory concentration (MIC) by witness.

The *asp*23 gene (LCAZH_0227) was selected as a target for mutational analysis because it was the most up-regulated protein in *L. casei* Zhang-G-1200 when the cells were grown in the presence of gentamycin. As shown in Figure 3, *asp23* inactivation did not affect the growth of *L. casei* Zhang-G-1200 in LSM without antibiotics. However, the *L. casei* Zhang-G-1200 *asp23* disruption mutant was significantly more sensitive to gentamycin with a relatively low MIC value of 8 μg/mL though still higher than that of the parental strain (vs. 2 μg/mL and 32 μg/mL for the parental strain and *L. casei* Zhang-G-1200, respectively). When the concentration of gentamycin increased to 16 μg/mL, the wild type *L. casei* Zhang-G-1200 cells survived significantly better than the *asp23* disruption mutant.

## Discussion

Probiotics that are used in food industry have low exposure to antibiotics normally. However, owing to their intrinsic antimicrobial property and desirable health-promoting effects, some scientists and clinicians have suggested using them to manage gastrointestinal disorders in clinical cases, when they may be directly exposed to antibiotics, sometimes even at a high concentration. Thus, it would be crucial to understand the evolutionary adaptation mechanisms of probiotics bacteria toward antibiotics as part of the risk assessment. In a previous long-term antibiotic-driven evolution experiment, we isolated the *L. casei* Zhang-G-1200 strain that exhibited elevated resistance to gentamycin compared with the parental line. The present study further characterized the mechanism of gentamycin resistance of this isolate using the iTRAQ-based proteomic approach.

*Lactobacillus casei* is a highly adaptable bacterium that can live on a wide range of niches; and it has a great capacity for choosing specific nutritional elements that enable its growth within any complex environments (Wang et al., 2012b). Like many other bacteria, it has developed sophisticated cellular mechanisms to regulate its nutritional responses. Carbon is one of the most important macronutrients for the bacterial cells; thus, its carbon metabolism and regulation have been studied in detail (Titgemeyer and Hillen, 2002). The strong ability *L. casei* in carbohydrate utilization relies very much on a rich array of PTSs present in its genome, ranking highest among all members within the *Lactobacillus* genus (Zhang et al., 2010). The current proteomic analysis revealed an apparent up-regulated expression of some PTS-related components in the gentamycin-resistant *L. casei* Zhang-G-1200 strain. The main function of PTSs is catalyzing sugar transport and phosphorylation (Zhang et al., 2013). The enhanced expression in *L. casei* Zhang-G-1200 may suggest an elevated cellular demand for utilizing different carbon substrates due to the presence of gentamycin.

Amino acid regulation is another important aspect required for sustaining growth of *L. casei*, especially for the late growth stages (Wang et al., 2012a). *L. casei* Zhang is able to synthesize most but the branch-chained amino acids; it is thus necessary for the bacterial cells to acquire the missing amino acids from the direct growth environment by proteolysis. *L. casei* Zhang possesses a well-developed proteolytic system (Wang et al., 2012b). In the gentamycin-containing environment, some of the key proteins involving in tryptophan and glutamate synthesis were found to be up-regulated, e.g., the tryptophan synthase alpha subunit and the glutamate synthase subunits. The tryptophan synthase alpha subunit functions to convert indole-3-glycerolphosphate into indole, the terminal step of tryptophan biosynthesis (Lim et al., 1991), while the glutamate synthase (large subunit) and NADPH-dependent glutamate synthase (small subunit) together catalyze the transamidation of the amide group from glutamine to 2-oxoglutarate to form glutamate (Stannek et al., 2015). Moreover, there were several up-regulated amino acid transporters and transporter-associated components, which might facilitate the intake of dipeptide/oligopeptides from the growth medium. The simultaneous up-regulation of multiple transporters for dipeptide/oligopeptides may suggest a need for *L. casei* Zhang-G-1200 to assimilate amino acids more efficiently under gentamycin stress.

Purine nucleotides are substrates for RNA and DNA synthesis; and they are essential for the growth of some LAB species (Wang et al., 2012a). Here, we also observed the up-regulation of some purine biosynthesis-related proteins. Among these proteins, the folate-dependent phosphoribosylglycinamide formyltransferase catalyzes the steps whereby the formyl derivatives of tetrahydrofolic acid are donated to the precursors of inosinic acid during its biosynthesis (Hartman and Buchanan, 1959); and the phosphoribosylformylglycinamidine synthase converts glycinamide ribotide into glycinamidine ribotide (Melnick and Buchanan, 1957). As in some other bacteria, the genome of *L. casei* Zhang contains a typical gene cluster for *de novo* purine biosynthesis, namely *PurCDFHKLM*, consisting of 12 distinct genes. The bacterial purine nucleotide synthesis is a 10-step pathway that produces inosinic acid from 5-phosphoribosyl 1-pyrophosphate (Ebbole and Zalkin, 1987). Since the culture medium used in the present study lacked purine compounds, the purine nucleotides must be obtained from the bacterial *de novo* biosynthesis, which was reflected by the up-regulation of these proteins.

Another group of differentially expressed proteins was the stress-related proteins. The molecular chaperones, such as *Gro*EL and *Gro*ES, play a central role in the control of general stress responses (Lemos and Burne, 2002). The *Gro*EL and several other chaperones of *L. casei* have been shown to be up-regulated in response to acid and bile stress (Wu et al., 2009b, 2010). Here, we observed a general reduction in expression of these proteins in the *L. casei* Zhang-G-1200 strain compared with its parental line under gentamycin stress except for the *Clp* protein and an alkaline shock protein (encoded by LCAZH_0227, the *asp*2 gene). These simply indicate that Zhang-G-1200 coped better in the antibiotics-containing environment due to its long-term adaptation to the drug. This was also reflected by a better growth performance of the adapted strain than its parental line in the presence of gentamycin.

It is interesting to note the high expression of *asp*23 in *L. casei* Zhang-G-1200 (21.9-fold increase compared with the parental strain). The activation of this gene is normally associated with alkaline shock conditions (Kuroda et al., 1995). To investigate its role in the gentamycin-resistant phenotype in the adapted strain, we created an *asp*23 disruption mutant. The gene disruption mutant appeared to be significantly more sensitive to gentamycin compared with the wild type *L. casei* Zhang-G-1200, though it was still more resistant than the parental *L. casei* Zhang, suggesting that this alkaline shock protein was partially responsible for the resistant phenotype. The bactericidal action of gentamycin resembles that of a ribosome modulation factor via the irreversible binding of the bacterial 30S ribosomal subunit and hence interrupting protein synthesis. Some stress-responsive proteins are also known to serve as ribosome modulation factors (Niven and EI-Sharoud, 2008). For example, the *E. coli RbfA* is a cold shock protein that regulates cold shock response by binding to the 30S ribosomal binding factor; its absence promotes cold shock responses (Jones and Inouye, 1996). Although the exact cellular role of *asp*23 remains to be determined, it is tempting to speculate that it acts as a modulator of similar nature and competes with gentamycin. However, the facts that the gentamycin-resistant phenotype of Zhang-G-1200 was developed stepwise along the long-term cultivation and only partial reversion of gentamycin-resistant phenotype was observed in the *asp23* disruption mutant together suggest that the resistant phenotype was not solely caused by the *asp23* gene but possibly along with certain secondary pleiotrophic mutations accumulated in the bacterial genome. To our knowledge, this work reports for the first time the involvement of alkaline shock proteins in gentamycin resistance.

## Conclusion

Several clinical trials supported the use of probiotics in the management of acute gastroenteritis and antibiotic-associated diarrhea, which elevates the chance of probiotics exposure to antibiotics. The present study characterized the mechanism of adaptive gentamycin resistance in a probiotic *L. casei* strain. We observed up-regulation in some carbohydrate, amino acid, and purine metabolic pathways in the resistant strain, which may function to support the bacterial growth under gentamycin stress. Meanwhile, some stress-related proteins were differentially expressed, particularly an alkaline shock protein encoded by the *asp*23 gene. The disruption of *asp*23 gene partially reversed the gentamycin-resistant phenotype, suggesting that this gene was involved in the resistance development although other accumulated mutations might play a role too. Normally, comparative proteomics should be performed in conditions, where the growth rates, cell densities and other culture parameters are as identical as possible between the strains that are compared. As the two strains studied here showed different growth performance in the presence of gentamycin, some changes in the proteome could result from the different physiological status between cultures and not directly from a specific differential response to gentamycin.

## Author contributions

WZ, ZS, and HZ designed the study. WZ, L-YK, and ZS wrote the manuscript. ZS, HG, CC, LL, and L-YK performed experiments. WZ and ZS analyzed data. All authors reviewed the manuscript.

### Conflict of interest statement

The authors declare that the research was conducted in the absence of any commercial or financial relationships that could be construed as a potential conflict of interest.
